# Genomic profiles of Indonesian colorectal cancer patients

**DOI:** 10.12688/f1000research.109136.2

**Published:** 2023-02-21

**Authors:** Murdani Abdullah, Sofy Meilany, Hidayat Trimarsanto, Safarina G. Malik, Ninik Sukartini, Firhat Idrus, Saskia A. Nursyirwan, Virly N. Muzellina, Rabbinu R. Pribadi, Amanda P. Utari, Hasan Maulahela, Ari F. Syam

**Affiliations:** 1 Division of Gastroenterology, Pancreatobiliary, and Digestive Endoscopy, Department of Internal Medicine, Faculty of Medicine, Universitas Indonesia - Dr. Cipto Mangunkusumo National General Hospital, Jakarta, 10430, Indonesia; 2 Human Cancer Research Center, Indonesian Medical Education and Research Institute, Faculty of Medicine, Universitas Indonesia, Jakarta, 10430, Indonesia; 3 Virology and Cancer Pathobiology Research Center, Faculty of Medicine, Universitas Indonesia - Dr. Cipto Mangunkusumo National General Hospital, Jakarta, 10430, Indonesia; 4 Eijkman Institute for Molecular Biology, Ministry of Research and Technology/National Research and Innovation Agency, Jakarta, 10430, Indonesia; 5 Department of Clinical Pathology, Faculty of Medicine, Universitas Indonesia - Dr. Cipto Mangunkusumo National General Hospital, Jakarta, 10430, Indonesia

**Keywords:** genetics, mutation, colorectal cancer, Indonesia, sequencing

## Abstract

**Background:** Colorectal cancer (CRC) is one of the most commonly diagnosed cancers worldwide and genetic mutation plays a vital role in CRC development. A previous study has suggested that genetic alterations among Indonesian patients with CRC might differ from those known in developed countries. This study aimed to describe the genomic profiles of Indonesian patients with CRC.

**Methods:** A total of 13 patients were recruited for this study from May to July 2019. Tissue samples were collected, and genomic DNA was extracted from the samples. AmpliSeq for Illumina Cancer HotSpot Panel v2 Next-generation sequencing was used for DNA sequencing and a genome analysis toolkit was used for local realignment around the discovered variants.

**Results:** A total of 45 genes comprising 391 single nucleotide variants (SNVs) with a depth >10 were observed. The genes with the most variants were STK11, SMAD4, EGFR, and ERBB4 and the genes with the most non-synonymous variants were SMAD4, TP53, FGFR3, CDKN2A, and STK11. Genes and SNVs in at least 90% of all samples consisted of 43 genes comprising 286 variants. Genes with the most non-synonymous SNVs were EGFR, SMO, FGFR3, TP53, STK11, CDKN2A. Genes related to the chromosomal instability pathway, such as TP53, SMAD4, KRAS, and APC, are also found in the analysis.

**Conclusions:** Our findings showed that all patients with CRC in this study had genetic mutations in the chromosomal instability pathway. Analysis of genetic mutation of Indonesian patients with CRC might be crucial for advanced targeted therapy and for better clinical outcomes.

## Introduction

Colorectal cancer (CRC) is one of the leading causes of cancer-related mortality worldwide. CRC is the fourth most commonly diagnosed cancer and the third most deadly cancer in the world for both sexes. ^1^ CRC has been responsible for 881,000 (9.2%) cases of cancer-related mortality worldwide. In Indonesia, the World Health Organization (WHO) placed CRC fourth in terms of the highest mortality burden caused by malignancies. The incidence and mortality rates of CRC have rapidly increased over the past decades due to environmental changes, such as sedentary lifestyles and increased lifespan. Several studies have shown that the 5-year survival rate of patients with CRC has remained at approximately 60% in the last decade. ^2^^,^^3^

There are some reported differences in CRC characteristics between Western and other populations. The prevalence of CRC in the Western population under the age of 50 years is around 2–8%. ^4^^,^^5^ Sehbai *et al.* reported that the incidence of CRC in Asian Indian and Pakistani populations under the age of 50 years was higher than that of white populations in the United States of America (USA). ^6^ Epidemiological data in Indonesia also showed that the proportion of patients with CRC under 40 years old was more than 30%. Other studies in developed countries have found that young-onset CRC is often associated with family history. However, in a previous study among young Indonesian patients with CRC, there was no positive family history. ^7^ Early onset CRC in developed countries showed several characteristics, such as localization in the ascending colon, low pathological stage, rare metastasis, and a better prognosis. By contrast, most young patients with CRC in the Indonesian population showed distal localization (rectum), a high clinical population, and poor prognosis. ^8^

Genomic instability, which allows the accumulation of numerous genetic mutations, is essential for CRC development. There are three pathways of genomic instability in CRC. The first pathway is the chromosomal instability (CIN) pathway, which consists of several gene mutations, including those in APC, KRAS, SMAD4, and TP53. The second pathway is the microsatellite instability (MSI) pathway, which is caused by defects in the nucleotide mismatch repair (MMR) mechanism and is represented by mutations in MSH2, MLH1, MSH3, PMS1, and PMS2. The third pathway is the inflammatory pathway, which involves the expression and activation of nuclear factor kappa-B (NF-κB) and COX-2. ^9^ In developed countries, the CIN pathway is conventionally found among sporadic CRCs, whereas the MSI pathway is found among younger patients with CRC. A study in Indonesia indicated that young Indonesian patients with CRC have defects in the DNA MMR system, which may promote MSI. However, when tested using BAT26, a surrogate marker of MSI, the frequency of MSI was very low and consistent with sporadic cancer features. Further testing of the same specimens using SMAD4 protein expression confirmed that neither young nor older Indonesian patients with CRC exhibited the MSI pathway. Another study in Indonesia also supported the hypothesis that inflammation may play a role in Indonesian patients with CRC. These results indicate that the molecular characteristics of patients with CRC in Indonesia may differ, anchored by pathways different from those previously found in the developed countries. ^8^^,^^10^

These highly heterogeneous results among populations require further investigation. The characterization of molecular subtypes, particularly among Indonesian patients with CRC, will lead to improved treatment selection and outcomes, such as molecularly targeted agents, often called precision medicine. In this study, we investigated the genomic profiles of patients with CRC in Indonesia using next-generation sequencing (NGS) analysis. NGS enables the identification of various genetic mutations that might be used further in the new era of targeted therapy among patients with CRC.

## Methods

### Ethical statement

This study was approved by the Research Ethics Committee of Universitas Indonesia, Jakarta (Ethical Approval Number KET-582/UN2.F1/ETIK/PPM.00.02/2019) and by the Research Division of Cipto Mangunkusumo National General Hospital, Jakarta (Research Permission Letter Number 1602/22/1670/2019). Written informed consent was obtained from all patients to participate in this study and publication of the patients’ details.

### Patients and clinical specimens

A total of 13 patients with CRC undergoing surgical resection of primary tumors at Cipto Mangunkusumo National General Hospital, Indonesia, were consecutively recruited from May to July 2019. Clinical data, including gender, age, cancer location, metastasis, and staging, were recorded from a structural questionnaire and histopathological results. Tissues were collected and separated for the determination of the clinical stage of cancer by histopathologists and for specimen collection. The tissues were then stored with 10% fetal bovine serum in Dulbecco’s Modified Eagle’s Medium (DMEM) with 1% antibiotics containing penicillin and streptomycin in liquid nitrogen until the DNA extraction process was performed.

### DNA extraction and quality control

DNA was extracted from the tissue using Quick-DNA ^TM^ Mini prep plus kit (Zymo Research) following the manufacturer’s protocol. Nucleic acid quantity and purity were assessed using a Qubit fluorometer (Invitrogen, Carlsbad, CA, USA) and a Nanodrop spectrophotometer (Invitrogen), respectively. Only samples with a concentration of 1.04–5.5 ng/μL with purity range of 1.7–1.9 passed the assessment and were subjected to sequencing.

### Amplicon sequencing and variant discovery

Sequencing libraries were generated using AmpliSeq for Illumina Cancer HotSpot Panel v2 following the manufacturer’s protocol, and 2 × 150 bp paired-end sequencing was performed on the Illumina MiSeq system. The variant discovery was performed following the GATK best practice. Briefly, the sequencing reads of each sample were aligned to the human reference genome GRCh38 (hg38) with Burrows–Wheeler Aligner version 1.61 (BWA, RRID:SCR_010910). As the AmpliSeq protocol was short amplicon sequencing, the PCR deduplication step was skipped. GATK v4 (GATK, RRID:SCR_001876) was used for local realignment around the variants. Variants calling of both single-nucleotide variants (SNVs) and indels were performed using the HaplotypeCaller tool from GATK, and the candidate variants were annotated using snpEff with the GRCh38.86 dataset. The following variants were defined as non-synonymous SNVs: stop-gain SNVs, stop-loss SNVs, and frameshift indels. Non-coding SNVs were defined as variants in the non-protein-coding regions of the genes, such as introns or untranslated regions.

## Results

Thirteen samples from colorectal patients were sequenced using AmpliSeq for Illumina Cancer HotSpot Panel v2. Table 1 shows the clinicopathological characteristics of the patients in this study. Among these patients, nine were male (69.2%) and four were female (30.8%), with the highest proportion of patients are aged 55–59 (23.1%). Early onset CRC defined as age below 50 which were 38.4% of our total samples. Regarding cancer location, nine patients (69.2%) were detected to have left-sided CRC, whereas the other four were on the right-sided colon. We used the terms left-sided and right-sided, based on the anatomical mark. Right-sided CRC is cancer of the caecum and the ascending colon up to the hepatic flexure, while left-sided CRC comprises cancer of the splenic flexure and the regions distal to the splenic flexure, including the rectum. ^11^

**Table 1.** Clinicopathological characteristics of the patients.

Characteristics	Cases (n = 13)
**Gender**	
Male	9 (69.2%)
Female	4 (30.8%)
**Onset**	
Early onset	5 (38.4%)
Late onset	8 (61.6%)
**Location**	
Left-sided colon	9 (69.2%)
Right-sided colon	4 (30.8%)
**Staging**	
I	2 (15.4%)
II	6 (46.2%)
III	2 (15.4%)
IV	3 (23.1%)
**Metastasis**	
Liver	3 (23.1%)
Lung	0 (0%)
Undetected (Stages I, II, III)	10 (76.9%)
Could not be measured (Stage IV)	0 (0%)

Based on the staging, we found that two patients, six patients, two patients, and three patients were on stage I, II, III, and IV, respectively. Among all the patients, three who were eventually on stage IV had liver metastasis. No metastasis was found in the other 10 patients (particularly for patients with stages I, II, and III).

**Figure 1.**
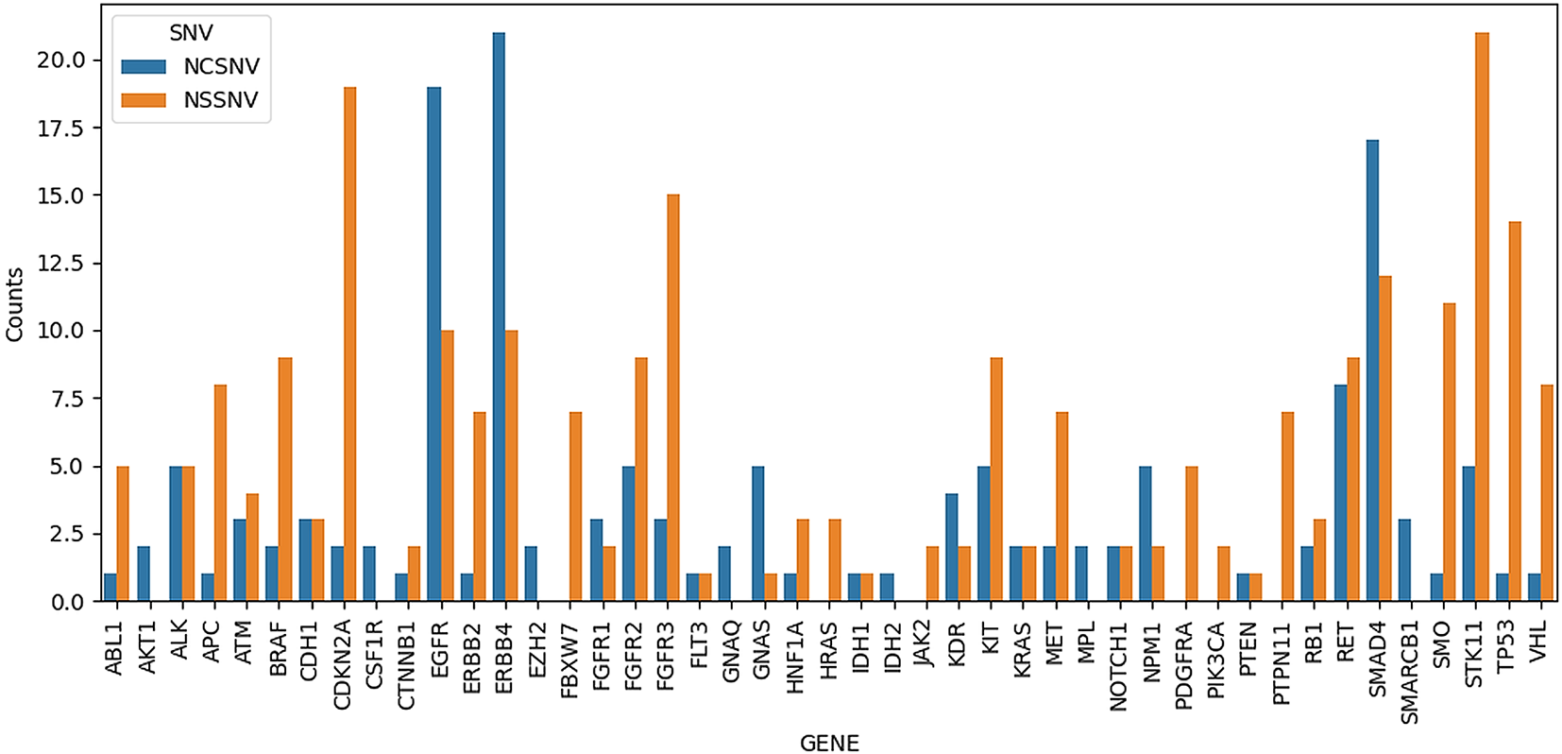
Plot of number of occurring single nucleotide variants (SNVs) in any of the samples. NCSNV denotes non-coding SNVs, while NSSNV denotes non-synonymous SNVs, which include missense, stop-gain, stop-loss, and frameshift variants.

**Figure 2.**
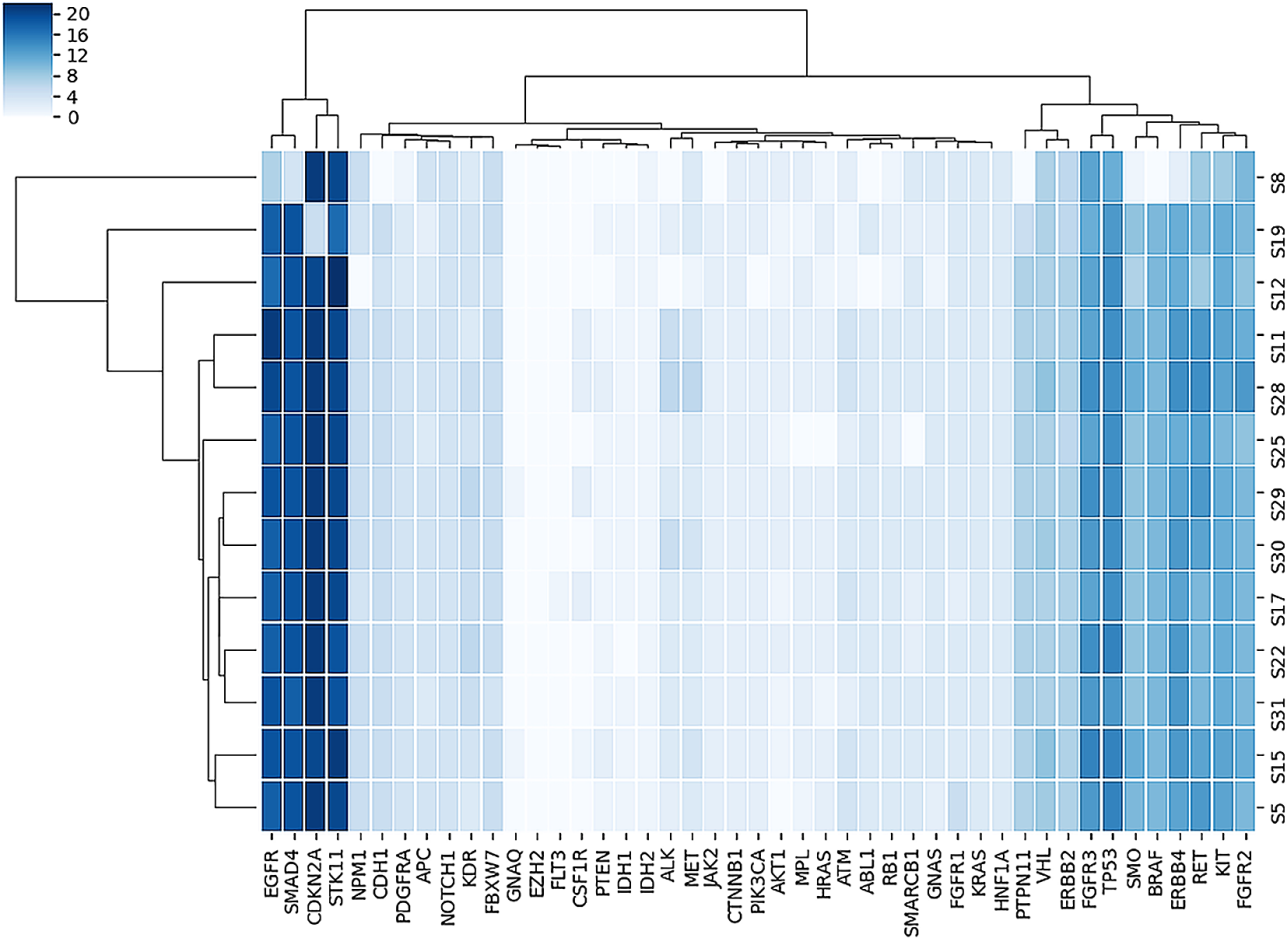
Plot of heatmap of number of non-synonymous variants observed in each gene for each sample.

Figure 1 shows all the SNVs that occurred in every sample with a depth > 10. A total of 45 genes comprising 391 variants were observed. The genes with the most variants observed were ERBB4 (31 SNVs), EGFR (29 SNVs), SMAD4 (29 SNVs), and STK11 (26 SNVs). Genes with the most non-synonymous variants were STK11, CDKN2A, FGFR3, TP53, and SMAD4 with 21, 19, 15, 14, and 12 SNVs, respectively. Figure 2 shows the heatmap of non-synonymous variants observed in each gene for each sample in this study.

Table 2 shows the gene details and SNVs that occurred in at least 90% of all samples, with a total of 43 genes comprising the 286 variants observed. Genes with the most non-synonymous SNVs were CDKN2A, STK11, TP53, FGFR3, SMO, and EGFR with 19, 16, 14, 12, 10, and 10 SNVs, respectively. It should be noted that genes related to the CIN pathway included APC, KRAS, SMAD4, and TP53, which had 5, 2, 4, and 14 non-synonymous SNVs, respectively ( Table 2).

**Table 2.** List of SNVs observed in at least 90% of samples with depth > 10.

Gene	Non-coding SNV	Non-synonymous SNV	Non-synonymous SNV amino acid changes
ABL1	0	3	Y345S N350T K375Q
AKT1	2	0	
ALK	0	5	A1200A L1198I G1202G S1206A K1205K
APC	0	5	S1504P A1470S T1556fs A1471G T1493T
ATM	1	3	A1732S N625T E1325V
BRAF	1	9	V600E S467* S465Y D454E P453P P453H I452V R444W L441V
CDH1	2	3	S337T F338L I374I
CDKN2A	2	19	D23H A22G A22P C21G D23A N20T E18D A17G G16G H15R L14P L12V L11V E10G V8G R7R S5G M3R M3V
CSF1R	2	0	
CTNNB1	0	2	A39S T40P
EGFR	9	10	M748I G765V Q742Q G530G L685L P688T P688Q E691D V693I Y824S
ERBB2	0	7	V853V V859G A867G D871E T875A Y877D A879G
ERBB4	5	8	G338V D335E A307P D300fs V298fs A235D R232R H231N
FBXW7	0	5	L403I T402N R385H R385C R425C
FGFR1	1	2	S35Y P34P
FGFR2	3	7	K85N P79H P75T F48I V41M N37K L148L
FGFR3	2	12	V266G L267V L267R V271G F273V V277G G753G H754L P755H P755P L760I L764P
FLT3	1	0	
GNAS	2	1	D229N
HNF1A	0	3	S256S L258R W267G
HRAS	0	2	H27H Q70E
IDH1	0	1	G105G
IDH2	1	0	
JAK2	0	2	V610G L611V
KDR	4	2	T1152T Q472H
KIT	3	9	K550N Y553D Y553* Q556K V559F V559G M541L K546K S25S
KRAS	1	2	G13D G12D
MET	1	3	S985G N375S S178S
MPL	2	0	
NOTCH1	2	2	L1678L G1673G
NPM1	3	2	D286E W290G
PDGFRA	0	4	N656N S692I S695R V824V
PIK3CA	0	2	A53T N114T
PTEN	0	1	R172S
PTPN11	0	7	P491P R498R E523G E523E L525R L525L Q526K
RB1	1	2	S758S R661R
RET	6	8	S904S A919S V648G L652R S653A S653S L769L S922S
SMAD4	15	4	F166F V258L P260T A367A
SMARCB1	3	0	
SMO	0	10	E194E L325L M326R G328G V329G R400G A401G F403L A406G V333V
STK11	5	16	S193P D194A D258H N259T P281Q A347S A347A D350Y D350E F354L D355E E357fs D358fs Y272* D284Y L182R
TP53	1	14	T253A C238Y P219P S185S R175H H168Y G154D V10I S9S G245S R273H S90fs R110R P72R
VHL	1	7	W117* W117* H125N T133I Q145K P146H R161*

## Discussion

In Indonesia, the prevalence of patients with CRC under 45 years of age was 47.85%. ^12^ The percentage of patients under 30 years old also increased significantly from 4.4% (2002–2006) to 9% (2007–2011). ^13^ These data are in concordance with those of a 25-year evaluation by Vuik *et al.*, who showed that the incidence of CRC was increased by 7.9% per year among subjects aged 20–29 years, 4.9% per year among subjects 30–39 years, and 1.6% per year among subjects 40–49 years in Europe. ^14^ The increasing dietary factors such as long-term consumption of alcohol and processed meat, lack of exercise, and obesity appear to be the possible causes for this increasing incidence. ^15^ Furthermore, urbanization and pollution are also associated with the overall increase in cancer incidence. ^16^

According to US data registries from 2009 to 2013, the lower gastrointestinal tract cancer distribution was more frequent in the proximal colon (proximal and including the splenic flexure, 41%), followed by the rectum (28%), distal colon (descending and sigmoid, 22%), and 8% in other sites. ^17^ It seems that our patients with CRC have different common locations of cancer. Tumors and cancers in the proximal colon and distal colon have several morphological and genetic differences. Flat sessile serrated adenomas and cancers are more common in the proximal colon than polypoid adenomas and cancers, which are more common in the distal colon. Distal colon tumors also more commonly present with chromosomally unstable tumors, ^18^^,^^19^ which is consistent with our finding that all subjects had genetic characteristics of mutation in the genes of the CIN pathway.

The next-generation sequencing (NGS) approach has been shown to be effective and accurate in determining the targeted therapy for cancer, including colorectal cancer. ^20^ This study focused on genetic mutations in Indonesian patients with CRC. We used Illumina Cancer HotSpot Panel, which covered 50 genes attributable to cancer. Our findings differed slightly from the data provided by the American Cancer Society (ACS), in which the CRC stage distribution among Asian/Pacific Inlander population was 37% local, 37% regional, 20% distant, and 7% unstaged. ^21^ We found more local and distant stage CRC, and fewer regional stage CRC. Halpern *et al.*, who investigated factors related to colon cancer stage at diagnosis, found that advanced-stage disease at diagnosis was common among uninsured patients, black patients, women, and patients from low socioeconomic status regions. Screening disparities may also lead to more advanced-stage colon cancer at diagnosis. ^22^

In this study, we found that KIT, KDR, TP53, ERBB4, APC, RET, and FLT3KI, which correlated with CRC development, were among the genes with substantial mutations in all 13 patients. These mutations were predicted to be somatic due to the absence of a family history of CRC among our patients.

KIT is a classic proto-oncogene and receptor tyrosine kinase that is activated through the PI3K, RAS, and JAK/STAT pathways. ^23^ These pathways are involved in tumor cell proliferation. KIT signaling is activated by the binding of its ligand, the stem cell factor (SCF) protein, which is activated by a phosphorylation cascade, resulting in the regulation of cell growth. ^24^

KDR is a gene that plays a role in stimulating blood vessel permeability and dilatation. Several studies have shown that KDR is a therapeutic biomarker that can be targeted by tyrosine kinase inhibitors. KDR also plays an important role in VEGF signaling, stimulating proliferation, chemotaxis, survival, and differentiation of endothelial cells. ^25^

We also observed a mutation in the TP53 gene. This gene is a tumor-suppressor gene and is associated with the progression of sporadic CRC. TP53 has many functions, such as DNA repair and cell cycle arrest, and it can trigger apoptosis when the damage is too severe. This gene mutation is associated with a poor prognosis due to the activation of the oncogenic and inflammatory pathways, which can accelerate CRC progression to later stages. ^26^

Activation of APC is a key process in β-catenin complex destruction. APC mutation leads to the accumulation of β-catenin protein in the cytoplasm and can promote the proliferation, migration, invasion, and metastasis of cancer cells. This gene mutation is found in 90% of patients with CRC. ^27^

ERBB4 is a member of the tyrosine kinase and EGFR sub-family, which promotes colonocyte survival. Activation of this gene can promote cellular responses, including proliferation, differentiation, apoptosis, survival, and migration of tumor cells. ERBB4 alteration is an early step in tumorigenesis, although the mechanism remains incompletely understood. ^28^

RET is a proto-oncogene that encodes transmembrane receptors with the tyrosine-protein kinase domain. The main function of RET is to induce apoptosis in cells through the regulation of several signaling pathways. ^29^ Mutation of RET results in kinase activation, which induces downstream signaling pathways such as PI3K, leading to tumor growth and cell survival. ^30^

FLT3KI is a gene that encodes a class III receptor tyrosine kinase that regulates hematopoiesis. This somatic mutation is commonly observed in patients with acute myeloid leukemia. Mutation of this gene leads to the activation of the FLT3 receptor tyrosine kinase and the proliferation of cells *in vitro.* ^31^

Unfortunately, the amplicon panel used in this study only covered genes related to the microsatellite instability pathway, MLH1. Similarly, genes related to inflammatory pathways were absent from this panel. Nevertheless, our findings clearly showed that all the patients with CRC presented here had genetic characteristics of mutation in the genes of the CIN pathway. We found mutations in KIT, KDR, TP53, ERBB4, APC, RET, and FLT3KI. This finding is different from our previous results, which showed COX2 expression among 49% of patients with CRC, NF-kB expression in 73.5% of the patients, and KRAS gene expression in only 16.3% of them. ^9^

TP53 and APC gene mutation in also similarly high in other countries such as Japan and US, although there are significant differences in mutation types between the two countries. ^32^ In China, KRAS, APC, and TP53 genes are also commonly mutated in CRC cases, along with CHEK2, MDC1, GNAQ, and SMAD4. ^33^ Similarly, these genes are also known to be frequently mutated among patients of African and European descent. However, there are differences such as KRAS and APC that are more frequent in African descent, whereas BRAF is less frequent compared to European descent. ^34^ A study in Australia found that POLE and POLD1 genes are associated with an increased risk of hereditary CRC, especially those who have the carrier genes. ^35^

The small sample size was a limitation of this study and could have affected the interpretation of the obtained results. However, this research was a pilot study that provided the first overview of gene mutations in Indonesian patients with CRC, due to the unavailability of data about Indonesian CRC gene mutations. The analysis of genetic mutations among patients with CRC might be important for future targeted therapy in CRC.

## Conclusions

Our pilot study of the genomic profile of patients with CRC showed that gene and SNVs in at least 90% of all samples consisted of a total of 43 genes comprising 286 variants, with most variants being STK11, SMAD4, EGFR, ERBB4. Genes with the most non-synonymous variants were SMDA4, TP53, FGFR3, CDKN2A, STK11. Our study found that all the patients with CRC had genetic mutations in the CIN pathway. Analysis of genetic mutation in Indonesian patients with CRC might be crucial for advanced targeted therapy and for better clinical outcomes.

## Data availability

### Underlying data

Open Science Framework: Genomic Profile of Indonesian Colorectal Cancer Patients. https://doi.org/10.17605/OSF.IO/JYPMA. ^36^

This project contains the following underlying data:

• Genomic Profile Raw Data.xlsx (This file contains clinicopathological data of patients recruited in this study)
• Genomic Profile Sequencing Data (This file contains raw genomic data of sequenced DNA from tissue samples)

Data are available under the terms of the Creative Commons Attribution 4.0 International license (CC-BY 4.0).
