# Supplementary material for: Genomic profiles of Indonesian colorectal cancer patients
Source: F1000Res. 2023 Feb 21;11:443. Originally published 2022 Apr 20. [Version 2] doi: 10.12688/f1000research.109136.2 (PMC10133825; doi:10.12688/f1000research.109136.2)
Supplement: Peer review history

# Peer review: Reviewer response for version 2

**DOI:** 10.5256/f1000research.144370.r164095

**Published:** 2023-04-26

**Authors:** Ahmad Rusdan Handoyo Utomo

**Affiliations:** 1 Graduate School of Biomedical Science, Universitas Yarsi, Central Jakarta, Jakarta, Indonesia

I would suggest to update Table 2 to include a column specifying numbers of pathogenic mutation. For examples BRAF V600E is pathogenic variant of SNV found in this cohort. So is G12D KRAS is also pathogenic SNV. These pathogenic SNVs should be tabulated in a column because their presence are known to be useful to guide clinical decision.

Is the work clearly and accurately presented and does it cite the current literature?

Partly

If applicable, is the statistical analysis and its interpretation appropriate?

Not applicable

Are all the source data underlying the results available to ensure full reproducibility?

Yes

Is the study design appropriate and is the work technically sound?

Partly

Are the conclusions drawn adequately supported by the results?

Partly

Are sufficient details of methods and analysis provided to allow replication by others?

Partly

Reviewer Expertise:

Cancer genetics, immunology

I confirm that I have read this submission and believe that I have an appropriate level of expertise to confirm that it is of an acceptable scientific standard.

# Peer review: Reviewer response for version 2

**DOI:** 10.5256/f1000research.144370.r164096

**Published:** 2023-02-22

**Authors:** Wei Zhang

**Affiliations:** 1 Department of Preventive Medicine, Northwestern University Feinberg School of Medicine, Chicago, IL, USA

The authors have revised the manuscript according to the prior review.

Is the work clearly and accurately presented and does it cite the current literature?

Partly

If applicable, is the statistical analysis and its interpretation appropriate?

Partly

Are all the source data underlying the results available to ensure full reproducibility?

Yes

Is the study design appropriate and is the work technically sound?

Partly

Are the conclusions drawn adequately supported by the results?

Partly

Are sufficient details of methods and analysis provided to allow replication by others?

Partly

Reviewer Expertise:

Cancer biomarker discovery, human genetics, bioinformatics

I confirm that I have read this submission and believe that I have an appropriate level of expertise to confirm that it is of an acceptable scientific standard.

# Peer review: Reviewer response for version 1

**DOI:** 10.5256/f1000research.120600.r150607

**Published:** 2022-09-20

**Authors:** Wei Zhang

**Affiliations:** 1 Department of Preventive Medicine, Northwestern University Feinberg School of Medicine, Chicago, IL, USA

Understanding genomic/genetic landscapes of CRC from different populations is critical for elucidating health disparities. The authors collected 13 tumor samples from Indonesian patients with CRC and generated genetic variant profiles using a candidate gene approach. The study design was straightforward and the manuscript was concisely written. The level of analysis and depth of description can be further improved though:

 Major comments:

 1. The results section is largely "direct" description of what was observed from the 13 tumor samples. It will be more interesting to add some description about comparisons for example early onset vs. late onset, early stages vs. late stages, as well as for example males vs. females, smokers vs non-smokers etc.

 2. The discussion section has a lot of redundant description of the results. The authors can improve the discussion by comparing with what is available in the literature for other populations (e.g., Eastern Asians, European Americans etc.).

 3. Also, the current discussion repeats too much about CRC epidemiology. The authors can try to be more focused by discussing their major findings as suggested in 2).

Is the work clearly and accurately presented and does it cite the current literature?

Partly

If applicable, is the statistical analysis and its interpretation appropriate?

Partly

Are all the source data underlying the results available to ensure full reproducibility?

Yes

Is the study design appropriate and is the work technically sound?

Partly

Are the conclusions drawn adequately supported by the results?

Partly

Are sufficient details of methods and analysis provided to allow replication by others?

Partly

Reviewer Expertise:

Cancer biomarker discovery, human genetics, bioinformatics

I confirm that I have read this submission and believe that I have an appropriate level of expertise to confirm that it is of an acceptable scientific standard, however I have significant reservations, as outlined above.

# Response

**Published:** 2023-02-10

**Authors:** Murdani Abdullah

**Affiliations:** University of Indonesia, Indonesia

We have revised the paper according to your comments. We have added the early onset and late onset proportions in our paper. We have also added comparisons of the mutated genes in our results with few literatures from different populations, namely US, China, European and African descendants, and Australians.

# Peer review: Reviewer response for version 1

**DOI:** 10.5256/f1000research.120600.r135274

**Published:** 2022-05-04

**Authors:** Ahmad Rusdan Handoyo Utomo

**Affiliations:** 1 Graduate School of Biomedical Science, Universitas Yarsi, Central Jakarta, Jakarta, Indonesia

The paper is describing numbers of genetic variants found in frozen tissues of 13 CRC patients undergoing surgical procedures. They found many genetic variants in many genes using Illumina NGS 50-gene panel. This is an interesting article providing preliminary results of genetic variants found using NGS. There are several issues that the authors need to address before the manuscript can be accepted for indexing.

 The authors should cite previous papers describing mutations in KRAS genes (Levi *et al.*, 2018 ^1^) and MIN pathways (Susanti *et al.*, 2021 ^2^) in Indonesia. The authors may discuss these papers and compare the results with the current manuscript.

 The methods should describe the percentage of tumor content of the specimens. This can be done by looking at the corresponding FFPE (formalin fixed paraffin embedded) block. This quality control check is to ensure that specimens being tested for NGS contained high percentage of tumor cells.

 The study design should describe the purpose of the study more clearly. The authors had described that indonesian colorectal patients may have different genetic and clinicopathological profiles (age, tumor locations) from western patients. Therefore, they should describe in their paper the prevalence of genetic mutations in different categories such as age and tumor locations as well. The paper should also analyse what is the percentage of transition and transversion mutations in order to gain insight and speculate the roles of local diet and pollutants. For instance the KRAS tranversion mutation has been associated with smoking (Dogan *et al.*, 2012 ^3^). There is no need for statistical analysis. A descriptive percentage table should be sufficient.

 The authors should also describe how many patients have pathogenic mutations of these genes since not all variants are pathogenic. KRAS G12D and BRAF V600E are certainly pathogenic mutations, but EGFR G765V is not. Describing the prevalence of clinically actionable genetic mutations using NGS may also demonstrate the utility of NGS in cancer genetic studies especially in developing countries like Indonesia.

Is the work clearly and accurately presented and does it cite the current literature?

Partly

If applicable, is the statistical analysis and its interpretation appropriate?

Not applicable

Are all the source data underlying the results available to ensure full reproducibility?

Yes

Is the study design appropriate and is the work technically sound?

Partly

Are the conclusions drawn adequately supported by the results?

Partly

Are sufficient details of methods and analysis provided to allow replication by others?

Partly

Reviewer Expertise:

cancer genetics, immunology

I confirm that I have read this submission and believe that I have an appropriate level of expertise to confirm that it is of an acceptable scientific standard, however I have significant reservations, as outlined above.

## References

[1] : Clinicopathological Associations of K-RAS and N-RAS Mutations in Indonesian Colorectal Cancer Cohort. *J Gastrointest Cancer* .2018;49(2) : 10.1007/s12029-016-9901-x 124-131 10.1007/s12029-016-9901-x 28044264

[2] : Molecular Analysis of Colorectal Cancers Suggests a High Frequency of Lynch Syndrome in Indonesia. *Cancers (Basel)* .2021;13(24) : 10.3390/cancers13246245 10.3390/cancers13246245 34944866PMC8699188

[3] : Molecular epidemiology of EGFR and KRAS mutations in 3,026 lung adenocarcinomas: higher susceptibility of women to smoking-related KRAS-mutant cancers. *Clin Cancer Res* .2012;18(22) : 10.1158/1078-0432.CCR-11-3265 6169-77 10.1158/1078-0432.CCR-11-3265 23014527PMC3500422

# Response

**Published:** 2022-06-03

**Authors:** Saskia Aziza

The study was designed to investigate potential biomarkers as biomarker candidates for Indonesia patients with colorectal cancer.

 A previous study on the clinical epidemiology of Indonesian patients with CRC found that the majority of patients were diagnosed between 45 and 50 years old, with a mean age around 47 years old. Immunohistochemical studies showed that patients with negative mismatch repair proteins were 43.5% for MSH2 and 83.5% for MLH1. ^1^ *KRAS *has been used as a potential tumor marker and its expression was reported to be as high as 30%-40% worldwide, but only 16.3% of 43 Indonesian patients with sporadic CRC cases have KRAS mutation and no BRAF mutation. This low KRAS mutation may indicate a different pattern of genetic instability in these colorectal tumors. ^1,2^ Along the sporadic colorectal carcinogenesis pathway, the cyclooxygenase-2 (COX-2) enzyme played a specific role during the polyp formation. COX-2 expression was reported in about 80% of CRC cases worldwide. ^3^ An Indonesian study found only 49% of COX-2 expression among CRC patients.

 Based on anatomical location, early-onset CRCs in developed countries most commonly occur in the rectum (35-37%), followed by the distal colon (25-26%) and proximal colon (22-23%) and showed a low pathological stage, rarely metastasize, and has a better prognosis. ^4,5^ However, Indonesian patients with CRC showed otherwise characteristics, i.e., commonly occur in the rectum (61.7%), distal (21.5%), proximal (16.7%), have a high clinical stage, and poor survival. ^1,6-7^

 The paper should also analyse what is the percentage of transition and transversion mutations in order to gain insight and speculate the roles of local diet and pollutants. For instance the KRAS tranversion mutation has been associated with smoking (Dogan *et al.*, 2012 ^3^ ). There is no need for statistical analysis. A descriptive percentage table should be sufficient.

 The percentage of transition and transversion mutation and the roles of local diet are indeed necessary and should be considered for our further study.
